# Cardiomyopathy Due to Nonsustained Ventricular Tachycardia Originating from the Aortic Sinus Cusp

**Published:** 2013-03-15

**Authors:** Hamidreza Bonakdar, Mohammad Assadian Rad, Jalal Kheirkhah, Anoush Barzigar

**Affiliations:** 1 Department of Pacemaker and Electrophysiology, Heshmat Cardiovascular Center, Guilan University of Medical Sciences, Rasht, IR Iran

**Keywords:** Electrophysiology, Radiofrequency Ablation, PVC

## Abstract

We report a case of idiopathic nonsustained ventricular tachycardia (VT) originating from the aortic sinus cusp referred for presyncope and LV dysfunction and frequent premature ventricular complex with no response to 3 months anti-arrhythmic medication for heart failure and arrhythmia. She was then referred to us for frequent PVC's and runs of nonsustained VT. ECG recorded during the nonsustained VT showed a left bundle branch block pattern in the precordial leads and an inferior axis and early transition in precordial leads in V3-V4. QS morphology in lead V1 was noticed with notching on the downward deflection. Electrophysiologic study was conducted to map ventricular outflow tract as a classic method, although pace map failed to find any matched QRS with the spontaneous PVCs. The mapping of aortic cusps was also performed. The best potential was recorded in a region located at the commissure of left-right aortic cusps. A single radiofrequency energy was delivered which resulted in immediate elimination of PVCs. The patient was discharged the day after ablation without any PVC recorded on monitor. Left ventricular ejection fraction(LVEF) improved to normal level two months later. There was no PVC detected at serial holter monitoring. It seems logical not to overlook even an isolated or nonsustained ventricular arrhythmia considering the available and effective treatments such as ablation rather than congestive heart failure(CHF) therapy especially in a young patient.

## 1. Introduction

Idiopathic premature ventricular complex (PVC) originating from the aortic sinus cusp is an uncommon finding in the clinical setting. The PVC can be abolished by radiofrequency (RF) catheter ablation, and in the case of ventricular tachycardia (VT), the mechanism is non-reentrant in most cases. Herein, we report a case of idiopathic nonsustained VT originating from the aortic sinus cusp.

## 2. Case Report

**Figure 1.**
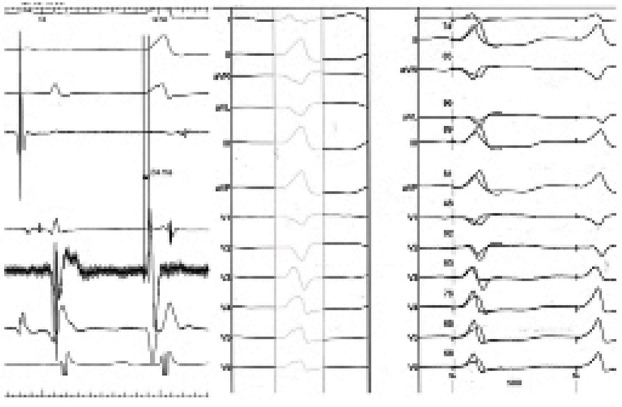
Morphology of PVC

A 24-year-old lady was referred for presyncope and LV dysfunction and frequent PVC. The patient's chief complaint was palpitation and exertional dyspnea since two years ago. Her last delivery was four years ago and she has been well at routine activities ever since. The exacerbation of symptoms occurred within recent three months. Echocardiography had revealed mild LV dysfunction (EF:40%). Surface ECG showed repetitive nonsustained monomorphic VT three months ago and her cardiologist began Sotalol to control her arrhythmia. The symptoms was not relieved after three month of treatment for heart failure and arrhythmia. She was then referred to us due to frequent PVCs and runs of nonsustained VT. ECG recorded during the non-sustained VT showed a left bundle branch block pattern in the precordial leads and an inferior axis and early transition in precordial leads in V3-V4 (Figure 1). This pattern fulfilled the criteria of VT originating from the aortic sinus cusp reported by Ouyang et al. (1); although this QRS morphology could be seen in PVC’s originating from Right ventricular outflow tract (RVOT)as well. Having obtained the informed written consent from the patient, she underwent Electrophysiologic study in fasting state, using the standard technique, with no antiarrhythmic agents. Quadripolar catheters were placed in the right ventricular apex and His bundle region via the right femoral vein. At first, using classic method, the right ventricular outflow tract was mapped, but pace map failed to find any matched QRS with the spontaneous PVCs. In addition the mapping of aortic cusps was performed. 

**Figure 2.**
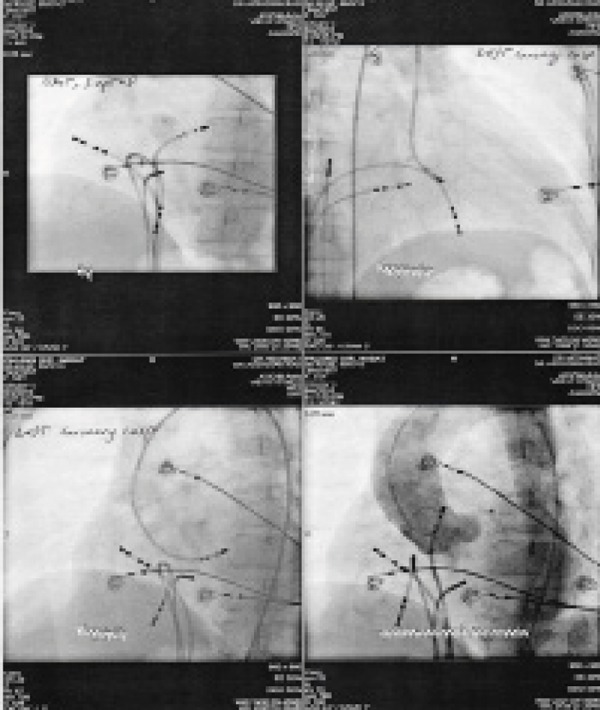
Activation and Pace Mapping

The finding of some good potential in left aortic cusp encouraged us to seek the origin of PVCs therein. A contrast aortogram was obtained to define the anatomy of aortic root and localization of coronary arteries. Great care was taken to avoid the ostium of the left main coronary artery, and mapping and ablation was only performed with stable catheter contact. The catheter of ablation was then placed retrogradely from aortic root and careful mapping was performed in left and right aortic cusps and their commissure. Meanwhile the (Activated clotting time) ACT was maintained above 250s to prevent clot formation. The best potential was recorded in a region located at the commissure of left-right aortic cusps (Figure 2). 

**Figure 3.**
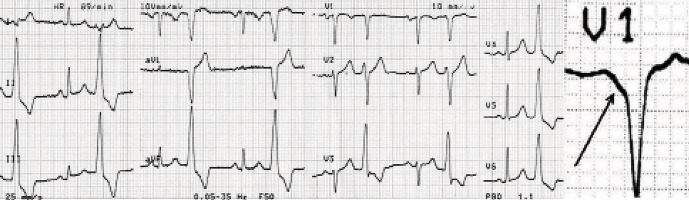
Views of Ablation Catheter

The recorded potential at the tip of the ablation catheter begins 24 msec earlier than the onset of PVC on the surface ECG which was quite suitable. In the commissure, however, morphology of pacing QRS through ablation catheter was not completely similar to patient’s PVC and was not of any help (Figure 2). The site was located more than 1.0 cm anteroinferior to the left coronary artery ostium in the left aortic sinus cusp (Figure 3). Regarding good earliest activation a single radiofrequency energy with setting 30W, 40Cwas delivered that resulted in immediate elimination of PVCs at less than 10 seconds. 

Following continued energy delivery up to 60 seconds VT was no longer inducible by RV extrastimuli or burst pacing before or after isoproterenol administration, and no PVC recurred during a 30- minute observation period in the EP lab. The patient was discharged the day after ablation without any recorded PVC on monitor. LVEF in echocardiography improved to normal level two months later, and no PVC was detected at serial holter monitoring.

## 3. Discussion

Tachycardia-induced cardiomyopathy has been associated with supraventricular and ventricular tachycardia and has been reported during recent decades (1). However, the mechanism by which isolated ventricular ectopy results in cardiomyopathy is not clearly defined. Guided by previous reports(2-8), we found the ECG characteristics of the PVCs to be extremely useful in localizing the site of origin of arrhythmia in our patient. This allowed efficient and rapid identification of the arrhythmia focus and ablation of the PVCs.

*3.1 Electrocardiographic Characteristics*

In our patient similar to the case reported by Strobel (9), we found four ECG characteristics useful in localizing the origin of arrhythmia: (1) An early precordial R wave transition; (3)The presence or absence of an S wave in leads V5 or V6; (8) Notch and slurring on the downslope of the QRS in lead V1 and (6)A rS in Lead I. The report from Kanagaratnam et al. (10)represents the patients with VTs arising from the sinus of Valsalva. All their 12 patients were reported to have a (Left bundle branch block)LBBB and inferior axis morphology with an early precordial transition, as described above. Nine of the 12 patients had VT arising from the left Sinus of Valsalva, whereas the other three patients had VT arising from the noncoronary sinus. The lead I morphology was useful in differentiating between the two groups. A rS complex was present in all patients with a left coronary sinus origin and a notched R wave was present in the patients with a noncoronary sinus origin. All patients had successful elimination of VT with radiofrequency ablation. Yamada et al, using aortic angiography, electroanatomic mapping, and limited echocardiography reported on five patients with ventricular arrhythmias originating from the junction of the left and right coronary sinus of Valsalva. They described a qrS pattern in leads V1–V3 from ventricular arrhythmias originating from the (left coronary cusp-non coronary cusp) LCC–RCC junction (11). Our result agrees with theirs, demonstrating the true specificity of this ECG finding.

Also, Bala et al. studied 37 patients with ventricular arrhythmia and noticed that the combination of a notch pattern in lead V1, precordial transition at lead V3, and the presence of late potentials in sinus rhythm at the site of successful ablation was identified in 8 of 19.

RCC–LCC ventricular arrhythmias, a finding not observed with any of the RVOT ventricular arrhythmias (12).

It has been reported that VT originating from the LVOT has a distinct ECG pattern, with a dominant S wave in lead I, right axis deviation, and an earlier precordial R wave transition zone (1,7). Hachiya et al. (3) had found that ablation for (left ventricular outflow tract) LVOT VT is often successful at the left coronary cusp if the S wave is absent in V5 and V6. ECGs of VT or PVC of the 11 patients in study of Hachiya all showed this characteristic with the only exception of an S wave in V5 of 1 patient, and all obtained successful ablation at the left coronary cusp. Moreover, we noticed QS morphology in lead V1 with notching on the downward deflection . Recently 37 patients were evaluated in terms of surface ECG and the reported results (12)were similar to our findings.

However, it is important to notice that pace mapping could not always be useful due to limitations like loss of capture at aortic cusp or not fully identical to spontaneous PVC as this case.

On the other hand, Duffee et al. (13)for first time reported 4 patients with more than 20'000 premature ventricular contractions over 24 hours and cardiomyopathy (ejection fraction <40%) with significant improvement in their left ventricular function after administration of drugs and complete abolition of PVC’s. Yarlagadda et al. (14) reported 8 patients with frequent RVOT PVC's and depressed LV function with complete recovery of LV function in 7 patients after successful ablation.

In our case, daily premature beats was about 9200 (about 10% of total count), which suggested that this less appreciated condition should be considered in any patient with unexplained cardiomyopathy with monomorphic ventricular ectopy. There is no data to explain why some patients develop cardiomyopathy in contrast with other patients (even with higher PVC count). Although in previous studies (1,7) the PVC's were mainly originated from RVOT, this important observation can be extended to P VC's originating from aortic cusps. About 20% of ventricular idiopathic arrhythmias with inferior axis morphology originate from aortic cusps, more frequently from left and right cusps and rarely from noncoronary cusp (15). Anatomic studies may provide the rationale for these findings (16, 17). Actually the NCC is adjacent to the atrial myocardium on the epicardial aspect and does not directly contact with myocardium of either the right or the left ventricle.

## 4. Conclusions

In patients with PVC and left bundle branch block and inferior axis morphology, the ECG is remarkably useful in rapidly localizing the site of origin for catheter ablation. An early transition zone in the precordial leads suggests a LVOT or aortic cusp site. Further, the absence of an S wave in leads V5 and V6 and slurring and notch on the downslope of the QRS in lead V1 suggests a site of origin within the sinuses of Valsalva. Finally, an rS complex in lead 1 is often present when the VT arises from the left sinus of Valsalva. On the other hand, the correlation of any arrhythmia and CHF should be considered at both sides and each one can be the potential cause of the other one. Determination of etiologic factor and choosing correct attitude is of great importance. It seems logical not to overlook even an isolated or nonsustained ventricular arrhythmia, considering the available and effective treatments such as ablation rather than CHF treatment especially in a young patient.
